# *Taenia solium* Cysticercosis Hotspots Surrounding Tapeworm Carriers: Clustering on Human Seroprevalence but Not on Seizures

**DOI:** 10.1371/journal.pntd.0000371

**Published:** 2009-01-27

**Authors:** Andres G. Lescano, Hector H. Garcia, Robert H. Gilman, Cesar M. Gavidia, Victor C. W. Tsang, Silvia Rodriguez, Lawrence H. Moulton, Manuel V. Villaran, Silvia M. Montano, Armando E. Gonzalez, and the Cysticercosis Working Group in Peru

**Affiliations:** 1 United States Naval Medical Research Center Detachment, Public Health Training Program (AGL) and Virology Program (SMM), Lima, Perú; 2 Johns Hopkins Bloomberg School of Public Health, Department of International Health, Baltimore, Maryland, United States of America; 3 Universidad Peruana Cayetano Heredia, School of Public Health and Administration (AGL and SMM) and School of Sciences, Department of Microbiology (HHG, RHG), Lima, Perú; 4 Instituto de Ciencias Neurológicas, Cysticercosis Unit, Lima, Perú; 5 Asociación Benéfica PRISMA (Proyectos en Informatica, Salud, Medicina y Agricultura), Research Department, Lima, Perú; 6 Universidad Nacional Mayor de San Marcos, School of Veterinary Medicine, Lima, Perú; 7 US Centers for Disease Control and Prevention, Division of Parasitic Diseases, Atlanta, Georgia, United States of America; George Washington University, United States of America

## Abstract

**Background:**

Neurocysticercosis accounts for 30%–50% of all late-onset epilepsy in endemic countries. We assessed the clustering patterns of *Taenia solium* human cysticercosis seropositivity and seizures around tapeworm carriers in seven rural communities in Peru.

**Methodology:**

The presence of *T. solium*–specific antibodies was defined as one or more positive bands in the enzyme-linked immunoelectrotransfer blot (EITB). Neurocysticercosis-related seizures cases were diagnosed clinically and had positive neuroimaging or EITB.

**Principal Findings:**

Eleven tapeworm carriers were identified by stool microscopy. The seroprevalence of human cysticercosis was 24% (196/803). Seroprevalence was 21% >50 m from a carrier and increased to 32% at 1–50 m (p = 0.047), and from that distance seroprevalence had another significant increase to 64% at the homes of carriers (p = 0.004). Seizure prevalence was 3.0% (25/837) but there were no differences between any pair of distance ranges (p = 0.629, Wald test 2 degrees of freedom).

**Conclusion/Significance:**

We observed a significant human cysticercosis seroprevalence gradient surrounding current tapeworm carriers, although cysticercosis-related seizures did not cluster around carriers. Due to differences in the timing of the two outcomes, seroprevalence may reflect recent *T. solium* exposure more accurately than seizure frequency.

## Author Summary

Cysticercosis is a parasitic disease caused by the tapeworm *Taenia solium*, common in areas with limited sanitation or with migration from these populations. The adult parasite is hosted in the human intestine and releases large numbers of eggs with the feces. Human beings sometimes ingest eggs due to poor hygiene, and then eggs sometimes lodge on the brain and after a few years can cause intense headaches and seizures. During a study in seven rural communities in Peru, individuals exposed to *T. solium* eggs were often tightly clustered at the homes or immediate surrounding of the carriers of the adult parasite. However, no aggregation of cases of seizures was found near carriers. It appears that seizures do not cluster around carriers because several years pass between exposure to *T. solium* eggs and the onset of seizures. During these years the adult parasite has probably died or people had moved within or even outside their communities. Therefore, only a partial understanding of the epidemiology of cysticercosis is gained by studying seizures cases.

## Introduction

*Taenia solium* cysticercosis is a parasitic disease endemic in developing countries where pigs are raised in close contact with human feces. Humans are the only definitive host and harbor the adult tapeworm. Taeniasis occurs after ingestion of improperly cooked pork and tapeworm carriers disseminate eggs in their feces. Cysticercosis is the infection with the larvae or cyst, and both people and pigs can become infected by fecal-oral contamination [1]. In humans, cysts often locate in the central nervous system (CNS) causing neurocysticercosis (NCC). Seizures are NCC's main clinical feature, although manifestations can range from asymptomatic, mild headaches and seizures to death [2]. Neurocysticercosis imposes a heavy financial burden to cases and their families, and treatment costs and productivity losses account on average for 53% of an annual minimum wage salary in the first year of treatment [3].

While it is well known that harboring a tapeworm or living with a carrier are factors associated with increased cysticercosis risk and disease burden [4],[5], it is not known if cysticercosis risk remains elevated outside the carrier's home. Two reports suggested that cysticercosis cases aggregate in neighboring households [6],[7], although they did not determine the magnitude and origin of clustering. In contrast, an epidemiological study published in 1992 actually did not detect clustering in human cysticercosis seroprevalence [8]. Recent work from our group demonstrated a substantial increase in swine cysticercosis seropositivity 50 and 200 m around tapeworm carriers and more extended seropositivity distance gradients [9]. However, given the different exposure patterns of pigs and humans, it remains unclear whether these findings can be extrapolated to human cysticercosis.

Quantifying the aggregation of human cysticercosis around carriers is a potentially relevant public health issue, because focalized control interventions could be developed to target such cysticercosis hotspots. We evaluated this hypothesis in northern Peru with the same methods previously used to assess clustering of swine cysticercosis. We determined if human cysticercosis also clustered around tapeworm carriers, comparing the clustering patterns of two key epidemiological parameters of human cysticercosis: seroprevalence and NCC-related seizures.

## Methods

### Study design and site

We analyzed data collected during the baseline assessments of a longitudinal study that evaluated control measures for *T. solium* cysticercosis. The study was conducted in seven rural, poor villages of the district of Matapalo, Tumbes, in Peru's northern coast near the Ecuadorian border. The study protocol was approved by the institutional review boards of the Universidad Peruana Cayetano Heredia, the Johns Hopkins Bloomberg School of Public Health and the Centers for Disease Control and Prevention. All study participants provided informed consent and assent if legal minors, using a single consent form was used for all study procedures. Prospective participants were told that they could refuse to participate in specific procedures. The socioeconomic characteristics of the area have been described elsewhere [9],[10]. We evaluated if there were increased rates of human cysticercosis seroprevalence and a lifetime history of NCC-related epileptic seizures surrounding tapeworm carriers.

### Taeniasis mass treatment and tapeworm detection

These procedures have been described in detail previously [9]. Briefly, *T. solium* taeniasis mass treatment took place between November–December 1999, together with a population census during which household coordinates were recorded with sub-meter accuracy using global positioning system (GPS) hand-held receivers. Eligible, consenting participants received a single dose of niclosamide (Pharmamed, Malta). Children under five and pregnant women were not treated, and treatment coverage was 95% among eligible residents. Stool samples were requested from all consenting residents before and after treatment regardless of age, pregnancy or treatment status. The study provided all the materials and instructions necessary to avoid self-contamination, and the stool collection rate among treated residents was 88%. Stool specimens were treated with an ether sedimentation technique and examined for *Taenia sp.* eggs by microscopy [11].

### Human serosurvey

During the census blood samples were taken by finger prick and stored on filter paper [12]–[14] to determine the presence of cysticercosis-specific antibodies with the serum enzyme-linked immuno-electrotransfer blot assay (EITB). Reactions to at least one of the seven diagnostic bands on EITB are considered positive. The EITB has 100% specificity, and its sensitivity is 98% in individuals with ≥2 viable lesions [15],[16] and approximately 60%–70% in patients with only one degenerating cyst or calcified lesions only [17].

### Neurologic evaluations

A validated questionnaire [18],[19] was applied to identify individuals with a history of epileptic seizures. Two different neurologists interviewed consecutively subjects selected by the questionnaire and confirmed or rejected the diagnosis following the criteria of the International League Against Epilepsy [20],[21]. Individuals with a confirmed diagnosis of epileptic seizures were offered a brain computed tomography (CT) scan to detect the presence of NCC-compatible lesions.

### Swine serosurvey

The swine serosurvey was conducted between November 1999 and January 2000, capturing pigs 2 months old and older excluding pregnant sows. A 6–8 ml serum sample was obtained by vena cava puncture and tested for cysticercosis-specific antibodies using the EITB assay.

### Statistical analyses

Two main outcomes were analyzed: 1) percent human cysticercosis seroprevalence and 2) lifetime prevalence of cysticercosis-related seizures, operationally defined for our analysis as seizure cases with either positive EITB or NCC-compatible lesions on the CT scan. Both outcomes were calculated at the individual level during univariate analyses and then aggregated by household during multiple regression analysis. The main covariate was the distance to the location of the nearest confirmed tapeworm carrier, calculated by assigning each subject the GPS coordinates of their household. Distances were calculated in meters using equator equivalences of latitude and longitude respectively [22]. The same procedures were used in our previous work [9].

We assessed the association between human cysticercosis and the distance to the nearest tapeworm carrier by separately estimating seroprevalence and lifetime seizure prevalence distance gradients using three different approaches. First, we described the shape of the distance gradient using piecewise cubic splines. Splines with 2–7 sections were initially defined with equal numbers of positive individuals in each section, and the best-fitting spline was then chosen. Second, we evaluated if seroprevalence or lifetime seizure prevalence increased exponentially near tapeworm carriers, testing if they were linearly associated with the base 2 logarithm of the distance [−log_2_(distance+1)]. Finally, seroprevalence and seizure prevalence rates were estimated for specific distance ranges. An additional analysis by distance ranges was conducted to evaluate the probability of finding tapeworm carriers in the proximity (50 m) of high seroprevalence households.

The human cysticercosis seroprevalence and lifetime seizure prevalence distance gradients were estimated using binomial family, logarithmic link function generalized linear models [23] to calculate prevalence ratios (PR). The relative change in prevalence rates associated to the distance to the nearest tapeworm carrier was estimated by:
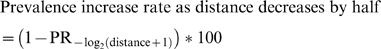



Statistical significance was determined using the Wald test and the Akaike's Information Criterion (AIC) was used to assess model fit. One degree of freedom (DF) Wald test are presented unless stated otherwise.

The association of the study outcomes with sociodemographic and swine farming covariates was also assessed. Neighborhood population density was assessed by the number of households in a 100 m radius. In-house crowding was measured with the ratio of household members per bedroom. Numeric variables such as neighborhood density, crowding, pigs owned and swine cysticercosis seroprevalence were categorized in tertiles. All these covariates were included in nested, sequential regression models to estimate adjusted seroprevalence and seizure prevalence gradients. Standard errors were scaled using the square root of the Pearson's χ^2^ dispersion parameter to account for the correlation in outcomes of individuals from the same household.

For further validation, two secondary outcomes were analyzed at the univariate level only: 1) seroprevalence defined by ≥3 positive EITB bands, as stronger EITB positivity is associated with greater parasitic burden and more severe infection [24],[25], and 2) lifetime overall seizure prevalence, either NCC or non-NCC related. All statistical analyses were performed using Stata 9.2 (Stata Corporation, US) and all confidence intervals (CI) were calculated at the 95% level. Maps were prepared with ArcMap 9.0 (ESRI, US).

## Results

### Census

The baseline census registered 1004 people and 237 families. We excluded 106 subjects from the analyses: 12 due to incomplete GPS measurements, 54 because they did not reside in the study area, and 40 temporary residents who stayed <2 nights per week in the area. Therefore, our analysis included 898 individuals from 212 families.

### Serological and stool surveys

**Figure 1:**
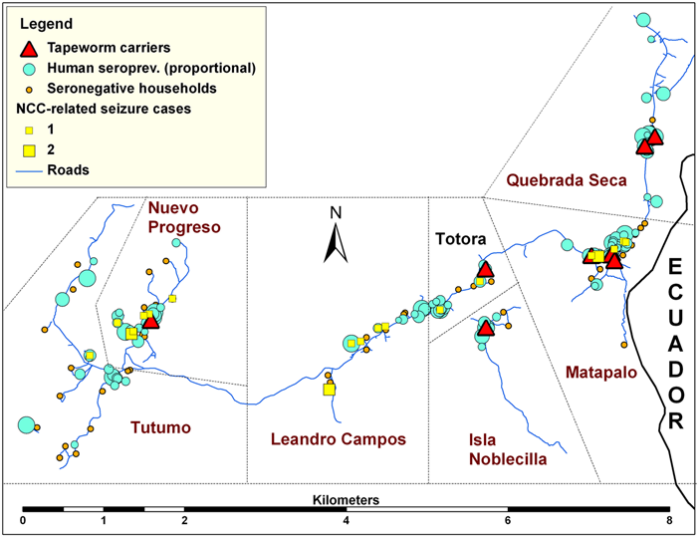
Proportion seroprevalence of human cysticercosis and location of *T. solium* tapeworm carriers and NCC-related cases, Tumbes, Peru, 1999. Two households with tapeworm carriers in Matapalo are virtually superimposed and cannot be differentiated visually. In the absence of defined community boundaries, straight lines were drawn to delimit villages respecting the self-reported area of residence data provided by each household.

The coverage of the serological survey was 89.4% (803/898) and the overall seroprevalence of cysticercosis was 24.4% (196/803), while 10.1% of all individuals had three or more positive EITB bands. Eleven tapeworm carriers were found in nine households during the stool survey, for a prevalence of taeniasis of 1.2%. The household seroprevalence of human cysticercosis and location of tapeworm carriers is presented in Figure 1.

### Seizure cases

The neurological survey reached 858 residents (95.6%) and 131 subjects reported a history of epileptic seizures. Two neurologists evaluated 111 of the 131 positive respondents and a random sample of negative responders who reported headaches or other neurological symptoms, confirming a lifetime history of seizures in 42 individuals (42/838, 5.0%). Only one tapeworm carrier reported a history of seizures, but was not confirmed during the neurological evaluation.

### NCC-related seizure cases

Out of 42 confirmed seizures cases, 23 were classified as NCC-related seizures because of NCC-compatible lesions on brain CT (15 cases), positive serology with negative CT (8 cases) or positive serology but without CT (2 cases). Using the operational definition proposed for our analysis, the prevalence of NCC-related seizures was 3.0% (25/837) after excluding one seronegative seizure case who did not presented for CT scan.

### Seroprevalence and seizure prevalence around carriers

**Figure 2:**
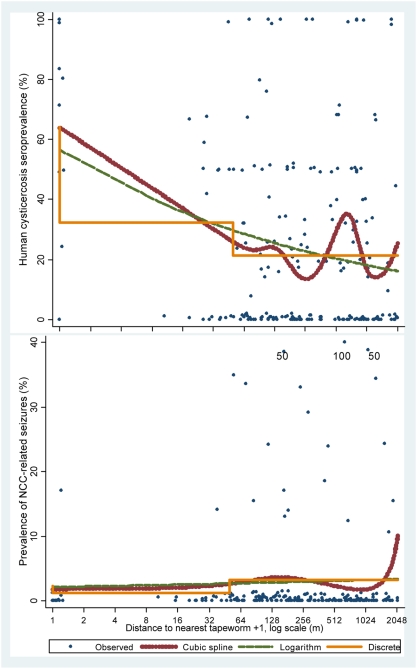
Household-level prevalence of human cysticercosis seroprevalence and NCC-related seizure by distance to the nearest *T. solium* tapeworm carrier, Tumbes, Peru, 1999. Observed prevalence rates in both figures are slightly jittered, and seizure frequencies 50% and 100% are represented as 40%.

Cubic splines showed increased human cysticercosis seroprevalence at the carriers' homes and in their immediate proximity (Figure 2, top). On average, seroprevalence had a 12% relative increase each time distance to the carrier halved (Table 1, 95% CI: 8%, 17%, p<0.001, Wald test). A statistically significant seroprevalence gradient was observed across distance ranges. Seroprevalence was 21% (148/693) >50 m from a carrier and increased to 32% (23/71) at 2–50 m (Prevalence ratio [PR] = 1.52, 95% CI: 1.01–2.29, Wald test p = 0.047), and from 1–50 m there was an additional increase to 64% (25/39) at the carriers' home (PR = 1.98, 95% CI: 1.25–3.14, Wald test p = 0.004). Human seroprevalence was marginally higher among carriers themselves compared to their household contacts (9/11 = 82% vs 16/28 = 57%, PR = 1.43, 95% CI: 0.93–2.21, Wald test p = 0.107). Distance to the nearest carrier fitted seroprevalence better using splines (AIC:2.31) compared to distance ranges (AIC:2.33) and logarithmic transformation (AIC:2.36).

**Table 1:** Covariate associations with the seroprevalence of human cysticercosis, Tumbes - Peru, 1999.

Variable	Positive	Tested	Prevalence	Prevalence ratio (PR)	p-value	Multivariate adjusted**		
					χ^2^	PR	95% CI	p-value***
**Village**					0.593			0.624
Tutumo	24	118	20.3	1.00		1.00	-	
Leandro Campos	18	83	21.7	1.07		1.09	0.59–2.03	
Nuevo Progreso	33	147	22.4	1.10		0.79	0.47–1.33	
Matapalo	59	239	24.7	1.21		0.79	0.45–1.39	
Isla Noblecilla	12	47	25.5	1.26		0.91	0.45–1.86	
Quebrada Seca	26	94	27.7	1.36		0.83	0.46–1.48	
Totora	24	75	32.0	1.57		1.14	0.62–2.08	
**Age (years)**					0.313			0.098
0–9	38	148	25.7	1.00		1.00	-	
10–19	41	199	20.6	0.80		0.74	0.49–1.10	
20–29	37	145	25.5	0.99		0.97	0.65–1.45	
30–39	33	131	25.2	0.98		0.95	0.62–1.44	
40–49	11	54	20.4	0.79		0.84	0.45–1.55	
50–59	22	61	36.1	1.40		1.50	0.96–2.36	
60+	14	65	21.5	0.84		0.71	0.42–1.19	
**Gender**					0.584			0.370
Female	88	347	25.4	1.00		1.00	-	
Male	108	456	23.7	0.93		0.89	0.69–1.15	
**Owns pigs** *					0.143			0.716
No	36	119	30.3	1.00		1.00	-	
Yes	146	611	23.9	0.79		0.93	0.63–1.38	
**# pigs** *					0.241			0.846
Doesn't own pigs	36	119	30.3	1.00		1.00	-	
1–3 (1st tertile)	36	169	21.3	0.70		0.84	0.51–1.36	
4–7 (2nd tertile)	55	201	27.4	0.90		1.00	0.64–1.56	
>7 (3rd tertile)	55	241	22.8	0.75		0.93	0.60–1.45	
**% seropositive pigs** *					<0.001			0.051
Doesn't own pigs	36	119	30.3	1.00		1.00	-	
< = 12.5% (1st tertile)	41	208	19.7	0.65		0.78	0.51–1.21	
13.3%–50.0% (2nd tertile)	42	226	18.6	0.61		0.72	0.47–1.10	
>50.0% (3rd tertile)	63	177	35.6	1.18		1.19	0.84–1.67	
**Households within a 100 m radius**					0.010			0.323
1–3 (1st tertile)	61	321	19.0	1.00		1.00	-	
4–8 (2nd tertile)	77	260	29.6	1.56		1.27	0.89–1.81	
>8 (3rd tertile)	58	222	26.1	1.37		1.06	0.73–1.56	
**Crowding (people/room)** *					0.896			0.256
0.3–1.2 (1st tertile)	29	132	22.0	1.00		1.00	-	
1.3–2.2 (2nd tertile)	63	278	22.7	1.03		1.07	0.72–1.60	
>2.2 (3rd tertile)	80	336	23.8	1.08		1.28	0.89–1.82	
**Latrine in household** *					0.881			0.360
No	125	520	24.0	1.00		1.00	-	
Yes	61	259	23.6	0.98		0.87	0.65–1.17	
**Distance to nearest tapeworm carrier decreases by half**	-	-	-	1.12	<0.001	1.10	1.05–1.15	<0.001
**Harbors a ** ***Taenia solium*** ** tapeworm**					<0.001			0.030
No	187	792	23.6	1.00		1.00	-	
Yes	9	11	81.8	3.47		1.71	1.05–2.77	
**Total**	196	803	24.4					

*: Information about pig rearing, crowding and latrine use was not obtained for all households.

**: Adjusted by the base 2 logarithm of the distance to the tapeworm and harboring a tapeworm.

***: Wald test.

In contrast to seroprevalence, there was no indication in any analyses of a trend in the frequency of NCC-related seizures associated to the distance to the nearest tapeworm carrier (Figure 2, bottom). Only two of the 25 cases of NCC-related seizure lived within 50 m of a carrier, and splines did not show any association between the distance to the nearest carrier and seizures, regardless of the number of curve segments fitted. No significant associations were observed in the analyses by the logarithm of distance (Wald test p = 0.639) and distance ranges (Wald test p = 0.629, 2 DF), respectively. No systematic overall trend was observed across distance ranges either (Table 2).

**Table 2:** Seizure frequency by distance to the nearest tapeworm carrier, Tumbes - Peru, 1999.

Distance to nearest tapeworm carrier	All seizures			NCC-related seizures		
	n / N	%	p-value	n / N	%	p-value
Carriers' home	2 / 42	2.4	0.604	2 / 42	2.4	0.629
2–50 m	2 / 80	2.5		1 / 80	1.2	
>50 m	38 / 716	5.3		23 / 715	3.2	
Tapeworm carriers	0 / 11	0.0	0.992	0 / 11	0.0	0.996
Household contacts	2 / 31	6.5		1 / 31	3.2	
**Total**	42 / 838	5.0		25 / 837	3.0	

In the analysis of secondary outcomes, seroprevalence defined by the presence of three or more positive EITB bands increased 13% each time distance to the nearest carrier halved (95% CI: 6%, 22%, Wald test p = 0.002). The seroprevalence distance gradient, however, was observed only within 50 m of carriers. There were no differences between >50 m and 1–50 m from a carrier (62/693 = 8.9% vs 5/71 = 7.0%, Wald test p = 0.637), but seroprevalence increased from 7.0% in 1–50 m to 14/39 = 35.9% at the carriers' home (PR = 4.01, 95% CI: 2.32–6.93, Wald test p<0.001). Additionally, carriers had significantly higher seroprevalence than their household contacts (8/11 = 72.7% vs 6/28 = 21.4%, PR = 3.39, 95% CI: 1.48–7.80, Wald test p = 0.004). The prevalence of all seizures, in contrast, was not associated to the distance to the nearest carrier, neither as the base 2 logarithm of the distance nor when analyzed by distance ranges (Wald test p = 0.842 and p = 0.604 [2 DF], respectively).

### Other variables associated with seroprevalence

Human cysticercosis seroprevalence was not associated with age, gender, village, number of pigs owned, crowding and availability of latrines. Families that owned pigs had a marginally lower seroprevalence, and human population density and seroprevalence in pigs were associated with significant differences too. In the only village where some households had sewage (Matapalo), human cysticercosis seroprevalence was positively associated with the presence of sewage installations. None of these variables, however, were significantly associated with human seroprevalence after adjusting for distance to the carrier and harboring a tapeworm. Human cysticercosis seroprevalence remained increasing by 10% as distance to the nearest carrier decreased by half (95% CI: 5%, 15%, Wald test p<0.001) after adjusting for having a tapeworm.

Despite a clear association between distance to the tapeworm carrier and human cysticercosis seroprevalence, the chance of finding a carrier 50 m around a high seroprevalence (≥80%) household never exceeded 31% in any of the models.

## Discussion

Our findings demonstrate a significant gradient in human *T. solium*-specific antibodies around tapeworm carriers, increasing from 21% at >50 m from tapeworm carriers to 64% at the homes of carriers themselves. Seropositivity hotspots and distance gradients around carriers have also been observed in swine cysticercosis [9], and gradients in *T. ovis and T. hydatigena* parasitic burden were also observed around canine carriers [26]–[28]. NCC-related seizures, however, did not show any clustering patterns surrounding carriers. Current or previous epileptic crises may not be an efficient, useful indicator to identify active cysticercosis transmission foci, and seroprevalence may reflect recent exposure more accurately than seizure frequency.

After a newly infected tapeworm carrier starts disseminating viable *T. solium* eggs and a seroprevalence gradient is formed around the carrier, several factors may prevent observing a carrier-centered seizure cluster later on. First, exposure does not always culminate in cyst implantation in the central nervous system and only a fraction of NCC cases will eventually present seizures [2]. Second, most tapeworms probably die before the 3–5 year NCC pre-patent period [29],[30], which leads to the relatively low taeniasis rates found in most symptomatic NCC patients and their relatives [5],[31], and suggests that NCC-related seizure cases found in the community are unlikely to coexist with the tapeworms that infected them. Third, seizure gradients around carriers will be confounded by the migration of carriers and epileptic individuals, other NCC-seizure cases infected by tapeworms outside the community and non-NCC related seizure cases. Therefore, the absence of seizure gradients around carriers may result because NCC-related seizure cases were probably infected mainly by previous generations of tapeworm carriers instead of currently present carriers. This means that when an NCC case first begins seizing, the tapeworm that infected him/her is at least three years old and most likely dead already. Alternatively, either the carrier or the NCC case may have moved farther from the case. Thus, the identification of risk factors for NCC-related seizures is probably clouded if covariates such as proximity to carriers are measured when symptoms onset or later instead of closer to the exposure period.

Close proximity to a *T. solium* tapeworm carrier is the main risk factor for swine cysticercosis seroprevalence [1], and we observed a strong association between seroprevalence and both harboring a tapeworm as well as the distance to the nearest carrier, even after multiple-regression adjustment. Other factors associated to seroprevalence in univariate analyses were not significant in regression analyses. The precarious latrines and sewage connections present in some villages [32] apparently failed to provide a protective effect, similarly to previous reports where the poor latrine design and maintenance probably did not reduce environmental contamination [9],[10]. Besides the proximity to a tapeworm carrier, most villagers of this uniformly rural area probably had similar chances of exposure to *T. solium* eggs.

Previous research demonstrated that the number of seropositive EITB bands is correlated to the number of NCC-related lesions observed in CT [24] and we found increased seropositive with three or more positive EITB bands at the homes of tapeworm carriers among other household members. Close, in-house contact with carriers may explain the stronger EITB responses observed, probably related to higher infective doses and increased parasitic burden [24]. However, increased seroprevalence with one or more positive EITB bands was found in subjects living up to 50 m from carriers. A significant fraction of EITB seropositive humans and pigs are known to serorevert [9],[33],[34], and in pigs this was more frequent in animals with 1–2 EITB bands [9]. Therefore, it is likely that most of the excess human seropositivity 50 m from carriers corresponds to subjects with transient immunity. Only a small fraction of the seroprevalent cases may actually develop cysts and could present seizures later, consistently with the rates of NCC-related lesions found in community settings [10].

Both swine and human cysticercosis seroprevalence presented significant gradients surrounding *T. solium* tapeworm carriers, although with certain differences. Higher human seroprevalence was observed at the carrier's home compared to other households <50 m of a tapeworm, but no such difference in swine seroprevalence was observed. Increased human cysticercosis seroprevalence was observed within 50 m from carriers, while swine cysticercosis remained increased up to 51–500 m [9]. Swine coprophagia and free-range pig rearing probably explain the extended swine seroprevalence gradient. Human transmission by fecal-oral contamination, in comparison, is probably a less-efficient exposure mechanism, concentrating increased exposure mostly in the immediate proximity of carriers.

A limitation of this study is the absence of application of a sensitive coproantigen ELISA for human taeniasis [35], a test that can detect twice as many tapeworm carriers compared to our Ritchie formol ether test [11]. Missed carriers reduced the statistical power of the analyses and probably introduced exposure misclassification, decreasing the chances of finding a true association between seizures and distance to the nearest *T. solium* carrier. However, the same eleven tapeworms detected were enough to show highly statistically significant cysticercosis gradients both in swine seroprevalence and seroincidence in a previous study [9] as well as in human seroprevalence in this study. The lack of a seizure gradient probably was not due to low statistical power, because with similar number of detected carriers and cases allowing demonstrating a distance seroincidence gradient in pigs but no seizure rate trends in humans.

The observed seroprevalence gradients provide a first estimate of the size of *T. solium* cysticercosis seropositivity hotspots around carriers at 200 m and 50 m for swine and humans, respectively. In comparison, increased *T. hydatigena* infectivity was found 80 m from carriers [26] and up to 175 m for *T. ovis* [27]. Ecological associations suggested transmission over larger distances [27],[28], although without compelling evidence. Despite the limited comparability of findings due to differences between species, hosts, transmission mechanisms, research methods and context, the available data appears to suggest that tapeworm carriers spread contamination over a few hundred meters at best, with greater impact within smaller (50–80 m) radius.

Our results showed that human cysticercosis seroprevalence clustered in the immediate surroundings of *T. solium* tapeworm carriers but seizure frequency did not. The absence of seizure clusters suggests that the location of seizure cases does not correlate with recent transmission hotspots and probably has little use for prevention. Also, these results suggest that risk factor for seizures should attempt to address the period when cases were exposed instead of the time when symptoms began years later. Further insight into other epidemiological parameters such as tapeworm lifespan, duration of environmental contamination, and egg dispersion mechanisms should enhance neurocysticercosis control in the underdeveloped settings where this disease is one of the main causes of acquired epilepsy.

## Supporting Information

Alternative Language Abstract S1 Translation of the Abstract into Spanish by Andres G. Lescano
(0.03 MB DOC)
