# Supplementary material for: Taenia solium Cysticercosis Hotspots Surrounding Tapeworm Carriers: Clustering on Human Seroprevalence but Not on Seizures
Source: PLoS Negl Trop Dis. 2009 Jan 27;3(1):e371. doi: 10.1371/journal.pntd.0000371 (PMC2625436; doi:10.1371/journal.pntd.0000371)
Supplement: Alternative Language Abstract S1 — Translation of the Abstract into Spanish by Andres G. Lescano (0.03 MB DOC) [file pntd.0000371.s001.doc]

RESUMEN

**Antecedentes y Objetivos**: La neurocisticercosis es responsible por 30-50% de los casos de epilepsia de inicio tardío en países endémicos. Nosotros evaluamos los patrones de agregación de la seropositividad humana cisticercosis por *Taenia solium* y convulsiones en portadores de tenia en siete comunidades rurales del Perú.

**Métodos**: La presencia de anticuerpos específicos para *T. solium* fue definida como una o más bandas positivas en el ensayo de electro-inmuno transferencia blot (EITB, siglas en inglés). Los casos de convulsiones relacionadas a neurocisticercosis fueron diagnosticados clínicamente y tuvieron resultados positivos en neuro-imágenes o por EITB.

**Resultados**: Once portadores de tenias fueron identificados por microscopía en heces. La seroprevalencia de cisticercosis humana fue 24% (196/803). La seroprevalencia fue 21% >50m de un portador y subió a 32% dentro de 1-50m (p=0.047), y de esa distancia la seroprevalencia tuvo otro incremento significativo hasta 64% en las casas de los portadores (p=0.004). La prevalencia de convulsiones fue 3.0% (25/837) pero no hubieron diferencias entre ningún par de rangos de distancia (p=0.629, Prueba Wald con dos grados de libertad).

**Conclusiones**: Nosotros observamos una significativa gradiente de seroprevalencia de cisticercosis humana alrededor de los portadores de tenia actuales, pero las convulsiones relacionadas a cisticercosis no estuvieron concentradas alrededor de los portadores. Debido a diferencias en el momento que ocurren estas dos condiciones, la seroprevalencia puede reflejar exposición reciente a *T. solium* con mayor precisión que la frecuencia de convulsiones.
